# Supplementary material for: The interactome of intact mitochondria by cross-linking mass spectrometry provides evidence for coexisting respiratory supercomplexes
Source: Mol Cell Proteomics. 2017 Dec 8;17(2):216–32. doi: 10.1074/mcp.RA117.000470 (PMC5795388; doi:10.1074/mcp.RA117.000470)
Supplement: Supplemental Data [file supp_RA117.000470_133922_0_supp_23978_fzffwf.zip › spectra_annotation/mito_spectra_annotation/431-1-4-1-4-1.pdf]

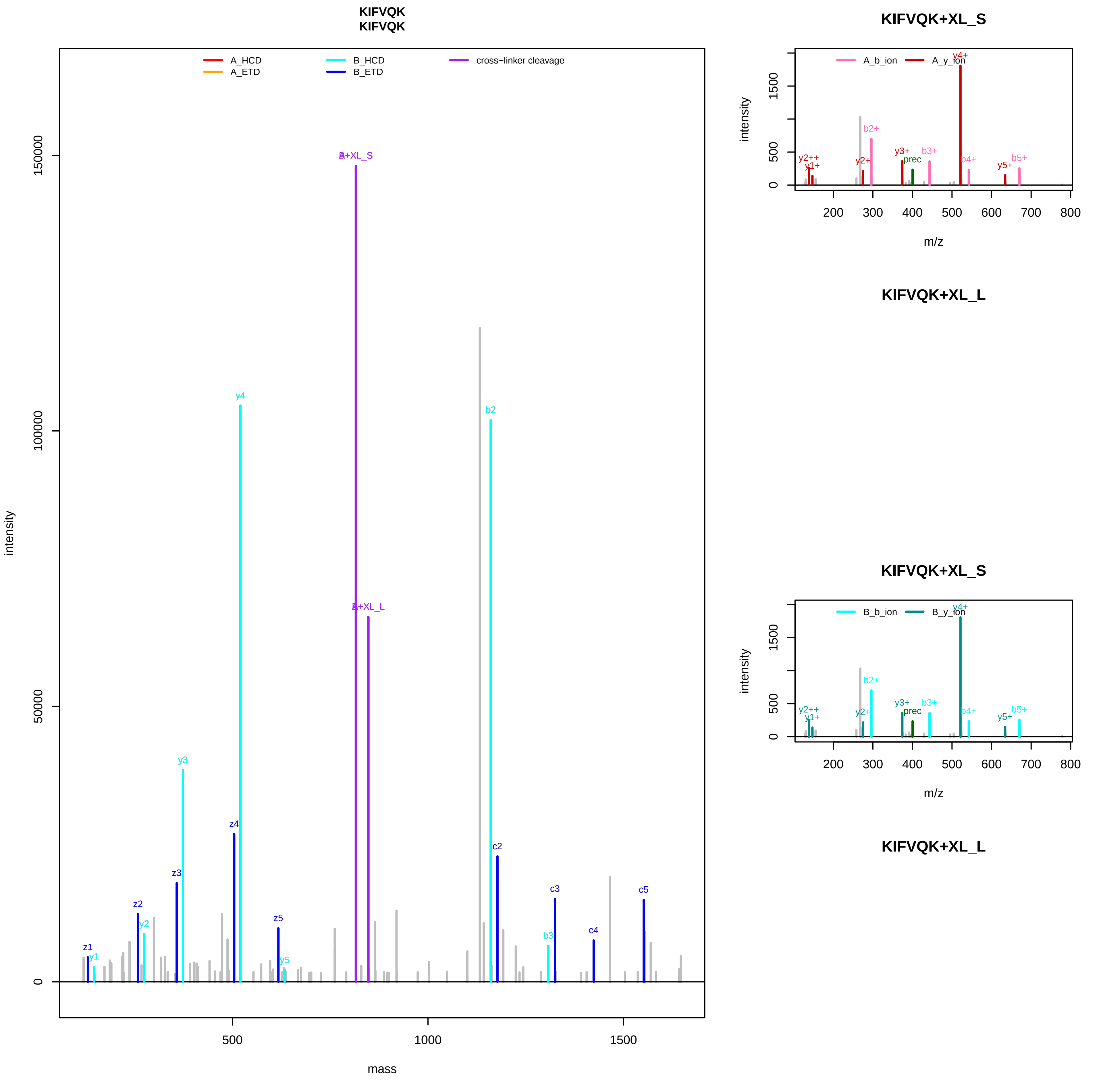
KIFVQK
KIFVQK
A_HCD
A_ETD
B_HCD
B_ETD
cross−linker cleavage
intensity
mass
0
50000
100000
150000
500
1000
1500
A+XL_S
A+XL_L
z1
y1
z2
y2
z3
y3
z4
y4
z5
y5
b2
c2
b3
c3
c4
c5
KIFVQK+XL_S
A_b_ion
A_y_ion
intensity
m/z
0
500
1500
200
300
400
500
600
700
800
y2++
y1+
y2+
b2+
y3+
prec
b3+
y4+
b4+
y5+
b5+
KIFVQK+XL_L
KIFVQK+XL_S
B_b_ion
B_y_ion
intensity
m/z
0
500
1500
200
300
400
500
600
700
800
y2++
y1+
y2+
b2+
y3+
prec
b3+
y4+
b4+
y5+
b5+
KIFVQK+XL_L
